# New Appliances and Things Medical

**Published:** 1905-01-07

**Authors:** 


### NEW APPLIANCES AND THINGS MEDICAL.

[We shall be glad to receive, at our Office, 28 & 29 Southampton Street, Strand, London, W.C., from the manufacturers, specimens of all new preparations and appliances which may be brought out from time to time.]

### COLLARGOLUM.

(Agents: Burgoyne, Burbidges and Co., 12 and 16 Coleman Street, E.C.)

Collargolum is a silver preparation soluble in water to the extent of 5 per cent. Its therapeutic value depends on its power of checking the processes of sepsis. Hence it may be applied locally to suppurating wounds and in cases of urethritis, vaginitis, etc. It has also been largely given internally in puerperal fever and other general septic conditions, and many experienced observers have found it of great value. The clinical evidence in its favour is very impressive.

### WORCESTERSHIRE SALT; "BLACK HORSE" BRAND.

(Salt Union, Limited, Droitwich.)

This is prepared from the celebrated Droitwich brine, and when dissolved in water can be used in the form of baths and packs in cases of chronic rheumatism and rheumatoid arthritis. A salt pack to the affected joints is an excellent local application in many cases of these diseases.

